# Ectopic Cdx2 Expression in Murine Esophagus Models an Intermediate Stage in the Emergence of Barrett's Esophagus

**DOI:** 10.1371/journal.pone.0018280

**Published:** 2011-04-06

**Authors:** Jianping Kong, Mary Ann Crissey, Shinsuke Funakoshi, James L. Kreindler, John P. Lynch

**Affiliations:** 1 Division of Gastroenterology, Department of Medicine, University of Pennsylvania, Philadelphia, Pennsylvania, United States of America; 2 Division of Pulmonary Medicine, Department of Pediatrics, The Children's Hospital of Philadelphia, Philadelphia, Pennsylvania, United States of America; Vanderbilt University Medical Center, United States of America

## Abstract

Barrett's esophagus (BE) is an intestinal metaplasia that occurs in the setting of chronic acid and bile reflux and is associated with a risk for adenocarcinoma. Expression of intestine-specific transcription factors in the esophagus likely contributes to metaplasia development. Our objective was to explore the effects of an intestine-specific transcription factor when expressed in the mouse esophageal epithelium. Transgenic mice were derived in which the transcription factor Cdx2 is expressed in squamous epithelium using the murine *Keratin-14* gene promoter. Effects of the transgene upon cell proliferation and differentiation, gene expression, and barrier integrity were explored. *K14-Cdx2* mice express the Cdx2 transgene in esophageal squamous tissues. Cdx2 expression was associated with reduced basal epithelial cell proliferation and altered cell morphology. Ultrastructurally two changes were noted. Cdx2 expression was associated with dilated space between the basal cells and diminished cell-cell adhesion caused by reduced Desmocollin-3 mRNA and protein expression. This compromised epithelial barrier function, as the measured trans-epithelial electrical resistance (TEER) of the *K14-Cdx2* epithelium was significantly reduced compared to controls (1189 Ohm*cm^2^ ±343.5 to 508 Ohm*cm^2^±92.48, p = 0.0532). Secondly, basal cells with features of a transitional cell type, intermediate between keratinocytes and columnar Barrett's epithelial cells, were observed. These cells had reduced keratin bundles and increased endoplasmic reticulum levels, suggesting the adoption of secretory-cell features. Moreover, at the ultrastructural level they resembled “Distinctive” cells associated with multilayered epithelium. Treatment of the *K14-Cdx2* mice with 5′-Azacytidine elicited expression of BE-associated genes including *Cdx1, Krt18*, and *Slc26a3/Dra*, suggesting the phenotype could be advanced under certain conditions. We conclude that ectopic Cdx2 expression in keratinocytes alters cell proliferation, barrier function, and differentiation. These altered cells represent a transitional cell type between normal squamous and columnar BE cells. The *K14-Cdx2* mice represent a useful model to study progression from squamous epithelium to BE.

## Introduction

Barrett's esophagus (BE) is a premalignant condition that increases the risk for esophageal adenocarcinoma [1], [2]. BE occurs at the gastroesophageal junction and is the replacement of normal squamous esophageal epithelium with an intestinalized columnar epithelium. It arises in the setting of chronic acid and bile reflux [3]. Despite its clinical importance, the molecular pathogenesis of BE is poorly understood. One limitation in the study of BE pathogenesis has been the lack of suitable experimental models. Most commonly, investigators have relied upon human BE tissue explants and esophageal adenocarcinoma cell lines for their studies [4], [5], [6], or primary esophageal keratinocytes and BE cell lines [7], [8], [9], [10], [11]. Current animal models require the surgical placement of either an esophagogastroduodenostomy or an esophagojejunostomy in rats and mice [12], [13], [14], [15]. Reflux esophagitis, metaplasia, and cancer have been described post-operatively. However, the response of mice to the surgery has not been as robust as with the rats [12].

An alternative approach is to utilize mouse genetics. This method was successfully applied in modeling gastric intestinal metaplasia [16], [17]. Transgenic mice were derived in which the *Caudal*-related transcription factor Cdx2 was ectopically expressed in gastric epithelium. Cdx2 is a transcription factor known to regulate intestine-specific gene expression, intestinal cell proliferation, and columnar shape, and is required for the normal intestinal epithelium development [18], [19], [20], [21], [22], [23], [24], [25], [26]. Gastric expression of Cdx2 induced an intestinal metaplasia consisting of goblet cells synthesizing intestinal mucins and absorptive cells expressing alkaline phosphatase. CDX2 is expected to play a similar role in the pathogenesis of BE [1], [27]. CDX2 mRNA and protein are uniformly expressed in BE biopsy samples, whether or not dysplasia is present [28], [29]. Moreover, CDX2 expression can be detected in the esophagus prior to the onset of BE, in the setting of chronic reflux esophagitis [28], [29], [30]. *In vitro* studies support this finding. Cdx2 expression can be induced in primary cultures of esophageal keratinocytes treated with short pulses of acid and bile [6], [31]. In the present study we demonstrate the effect that this ectopic Cdx2 expression can have upon squamous epithelium.

Using the *Keratin-14* gene promoter, we induce Cdx2 mRNA and protein expression in the basal cell layer of squamous epithelium. Cdx2 expression had significant effects upon cell proliferation, cell-cell adhesion, and basal cell morphology. Most significantly, Cdx2 expression promoted the adoption of a secretory-cell ultrastructure with increased endoplasmic reticulum levels and ER-associated proteins. These cells appear to represent an intermediate stage of differentiation between keratinocytes and columnar BE cells. Lastly, treatment of the *K14-Cdx2* mice, but not controls, with DNA methyltransferase inhibitors lead to the expression of intestine- and BE-associated genes. We conclude that the K14-Cdx2 mice represent an interesting mouse genetic model of early events in BE pathogenesis. Ectopic Cdx2 expression in the esophagus can initiate cellular changes consistent with an intermediate stage between normal squamous epithelium and an intestinalized, secretory epithelium similar to normal intestine and BE. Moreover, advancement to a more intestinalized metaplasia in the K14-Cdx2 mouse depends on changes in epigenetic gene regulation in the esophagus.

## Results

### The *Keratin-14* gene promoter directs expression of Cdx2 mRNA and protein to basal keratinocytes of squamous epithelium

**Figure 1:**
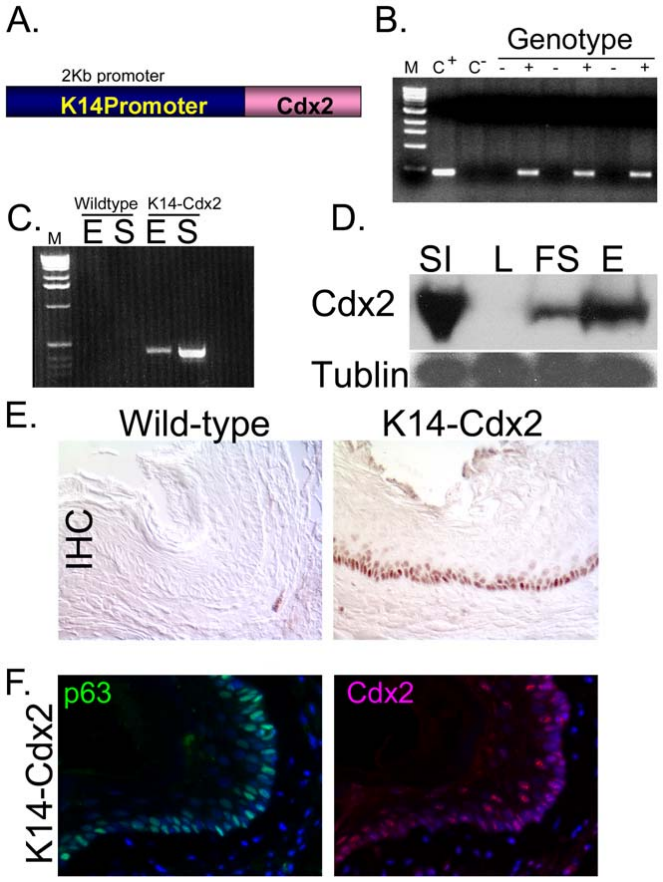
Generation of *K14-Cdx2* mice and pattern of transgene expression. **A** Schematic map of the transgene. **B.** PCR detection of the transgene in genomic DNA. **C+:** positive control, **C-;** negative control, **M**: size markers. **C.** Transgene expression by reverse-transcriptase PCR in RNA from esophagus (**E**) and Skin (**S**). **D.** Western blot for Cdx2 expression in whole cell lysates from small intestine (**SI**), liver (**L**), forestomach (**FS**), and the esophagus (**E**). Tubulin served as loading control. **E.** Immunohistochemical staining for Cdx2 expression in wild-type and transgenic esophagi. **F.** Immunofluorescence for Cdx2 (**Red**), p63 (**Green**), and DAPI (**blue**) in adjacent serial sections from transgenic esophagi.

Ectopic expression of Cdx2 using the *FoxA3* or the *H^+^/K^+^-ATPase* gene promoters was sufficient to induce a gastric intestinal metaplasia in transgenic mice [16], [17], [32]. To determine if a similar approach would yield a novel model for Barrett's esophagus, we subcloned a murine Cdx2 cDNA into a well-established K14/hGH transgenic expression vector (kindly provided by Elaine Fuchs, Rockefeller University) (Figure 1A) [33]. This vector reliably directs transgene expression to the basal keratinocytes of the squamous epithelium including the skin, tongue, esophagus, and forestomach. We established 7 transgenic lines bearing the *K14-Cdx2* vector. Two of the seven expressed transgene mRNA detected by quantitative PCR analysis, but only one line expressed Cdx2 protein observable by immunohistochemistry and Western blot analysis of nuclear extracts (Figure 1 and data not shown).

In the *K14-Cdx2* mice, ectopic Cdx2 expression was detected in the skin as well as the esophagus and forestomach (Figure 1C and D and Figure S1A and B). The level of Cdx2 expression in the esophagus was about 10% of that observed in the intestine, and the expression in the forestomach was even less, based on western blot comparison of whole-cell lysates from these epithelia and the liver as a negative control (Figure 1D). As expected based on previous experience with this K14 promoter construct [34], we observed Cdx2 protein expression predominantly in the nuclei of basal cells, with weaker expression in suprabasal cells (Figure 1E). As the squamous epithelium stem cells are reported to reside in this same basal cell layer [35], we stained by immunofluorescence for p63, a marker for these stem cells. Serial sections of esophageal tissue stained for Cdx2 or p63 suggest there is an overlap in their expression and therefore that the transgene Cdx2 is expressed in p63+ esophageal stem-cells (Figure 1F).

### Ectopic Cdx2 expression in esophageal keratinocytes significantly reduces cell proliferation

**Figure 2:**
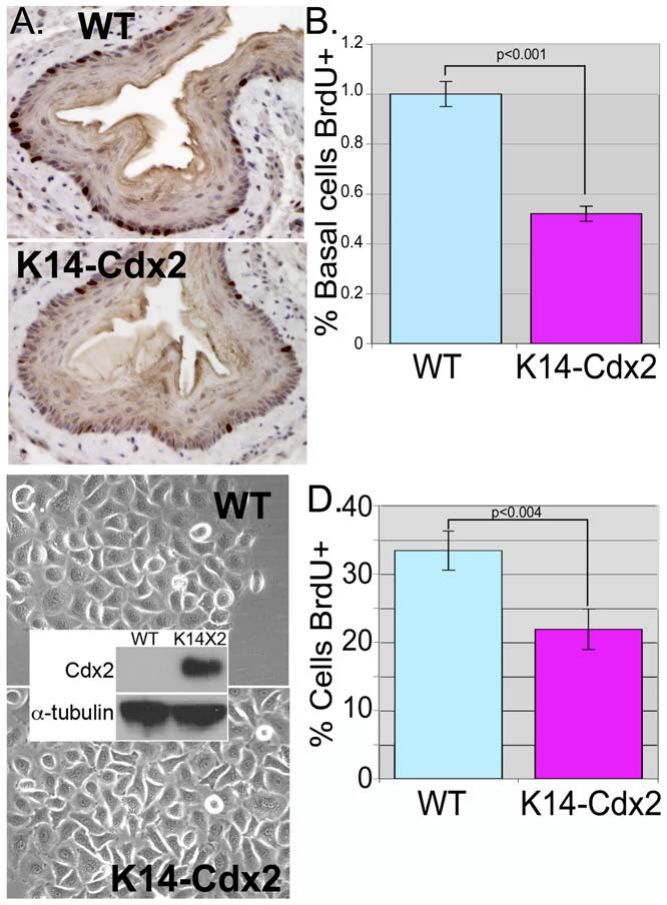
Cdx2 expression reduces cell proliferation of murine keratinocytes *in vitro* and *in vivo*. 3-month-old transgenic and wild-type littermates were injected intraperitoneally with BrdU 1 hour prior to sacrifice. **A.** Immunohistochemistry with Anti-BrdU antibody (US Biological) shows reduction in proliferating cell numbers in transgenic mice when compared with controls. **B**. Quantification of epithelial proliferation based on BrdU incorporation in the esophagus. BrdU positive cells per 150 basal epithelial cells were counted in four 3-month old *K14-Cdx2* mice and four matched littermate controls (n = 4). **Blue Bar**: Wild-type mice. **Red bar:** *K14-Cdx2* transgenic mice. **C.** Phase-contrast images of primary esophageal keratinocyte cell cultures established from transgenic and wild-type littermate mice shows similar morphology. **Inset**: Western blot for Cdx2 expression in nuclear lysates from primary cultured keratinocytes from K14-Cdx2 and wild-type mice. alpha-tubulin served as loading control. **D.** Cells were incubated with BrdU for 1 hour, then fixed and imaged for BrdU incorporation with DAPI counterstain. BrdU+ cells were counted in at least 100 total cells from 3 different primary transgenic cell lines and three littermate control lines. (n = 3).

Previous *in vitro* studies by us using a human esophageal keratinocyte cell line found that Cdx2 expression significantly reduced human keratinocyte cell proliferation [11]. Similarly, the Cdx2 transgene appeared to significantly diminish BrdU incorporation by proliferating keratinocytes *in vivo* (Figure 2A and 2B). BrdU incorporation rates per basal cell were half that of wild-type (wt) littermates. Apoptotic rates were not noticeably altered by the transgene (Figure S1C and 1D). Despite the measured difference in proliferation rates, we were able to establish *ex-vivo* cultures of esophageal keratinocytes from both wild-type and transgenic mice (Figure 2C). Expression of the transgenic Cdx2 protein is maintained in the *K14-Cdx2* cells even after several cell passages (Figure 2C, Inset). Consistent with the *in vivo* findings, the proliferation rate for the cultured transgenic keratinocytes was reduced by a third compared to wild-type control cells (Figure 2D). This suggests that diminished proliferation of the *K14-Cdx2* keratinocytes is largely due to effects upon the keratinocytes, since the reduction is maintained when cells are cultured under identical conditions *ex vivo*.

### Cdx2 expression alters basal cell morphology and ultrastructure in the esophagus and forestomach

**Figure 3:**
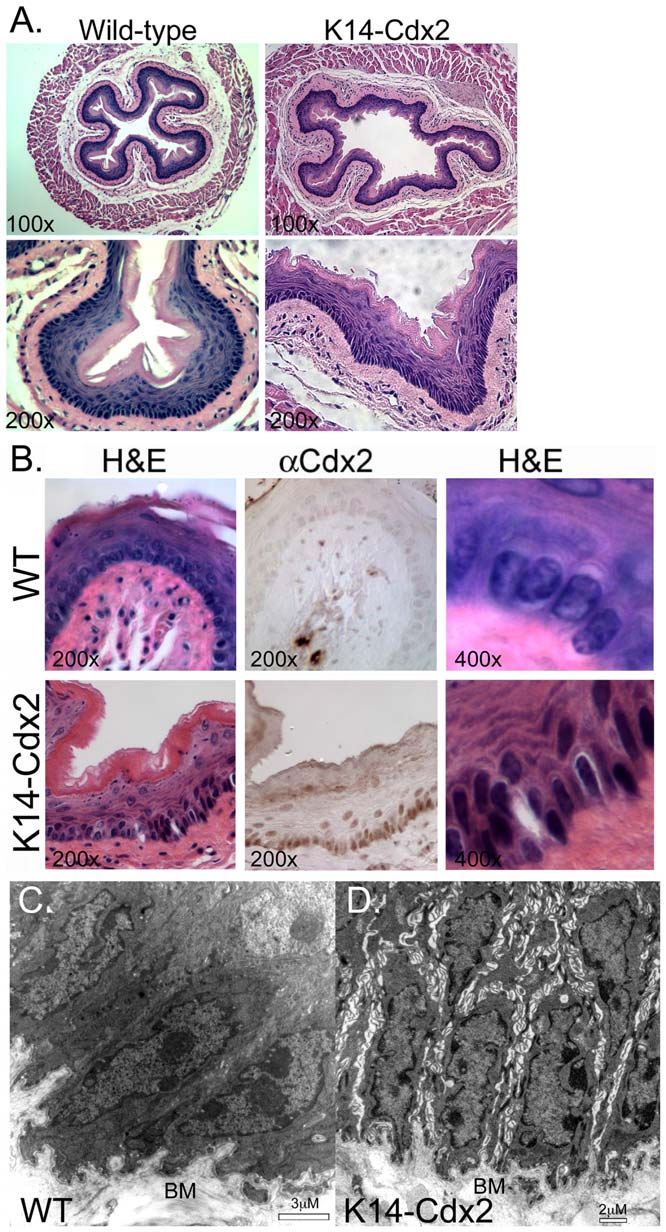
Transgene expression alters basal epithelial cell morphology and ultrastructure. **A.** Hematoxylin and eosin (H&E) staining of 3 month-old wild-type and transgenic esophagi tissue at low-power. **B.** Higher-powered images of serially-sectioned tissues stained either with H&E or with the Cdx2 antibody. Basal cell layer changes in cell morphology, in particular crowded, elongated nuclei can be observed. **C–D.** Transmission-electron microscopy (TEM) images of wild-type and transgenic esophageal tissue. **BM** = basement membrane.

Examinations of the esophagus at three months revealed no obvious defects in tissue architecture of the *K14-Cdx2* mice (Figure 3A). Despite the reduced cell proliferation, *K14-Cdx2* epithelium appeared to be of normal thickness with a differentiated and cornified superficial layer. However, on higher power examination, we noted subtle changes in cell morphology of the basal cell layer. Nuclei of the *K14-Cdx2* basal keratinocytes appeared to be elongated and the cells more densely packed and disorganized than in the wild-type epithelium (Figure 3B).

To better appreciate these morphological changes, we examined the tissue ultrastructure by transmission electron microscopy (TEM). Esophagi from 3 month old *K14-Cdx2* transgenic and wild-type control littermates were excised, fixed, and examined by TEM. We noted a number of changes in our transgenic mouse esophageal basal cells when compared to controls. In the controls, basal keratinocytes appeared rectangular to cuboidal, with dark cytoplasms full of electron-dense keratin bundles (Figure 3C). Basal cells appeared to be strongly adherent with adjacent cells, as evidenced by the many desmosomal junctions and little unfilled space between cells.

In contrast, transgenic mouse basal cells were very different. Basal cells with electron-dense cytoplasm and keratin bundles seemed more elongated than in the wild-type animals. Moreover, the epithelium had the appearance of pseudo-stratification, with many cells in the suprabasal region extending long cellular processes to the basal lamina (Figure 3D). The purpose of these long processes was unclear, but their presence suggests cells reluctant to disengage from the basement membrane as would be normal for upward-migrating keratinocytes.

**Figure 4:**
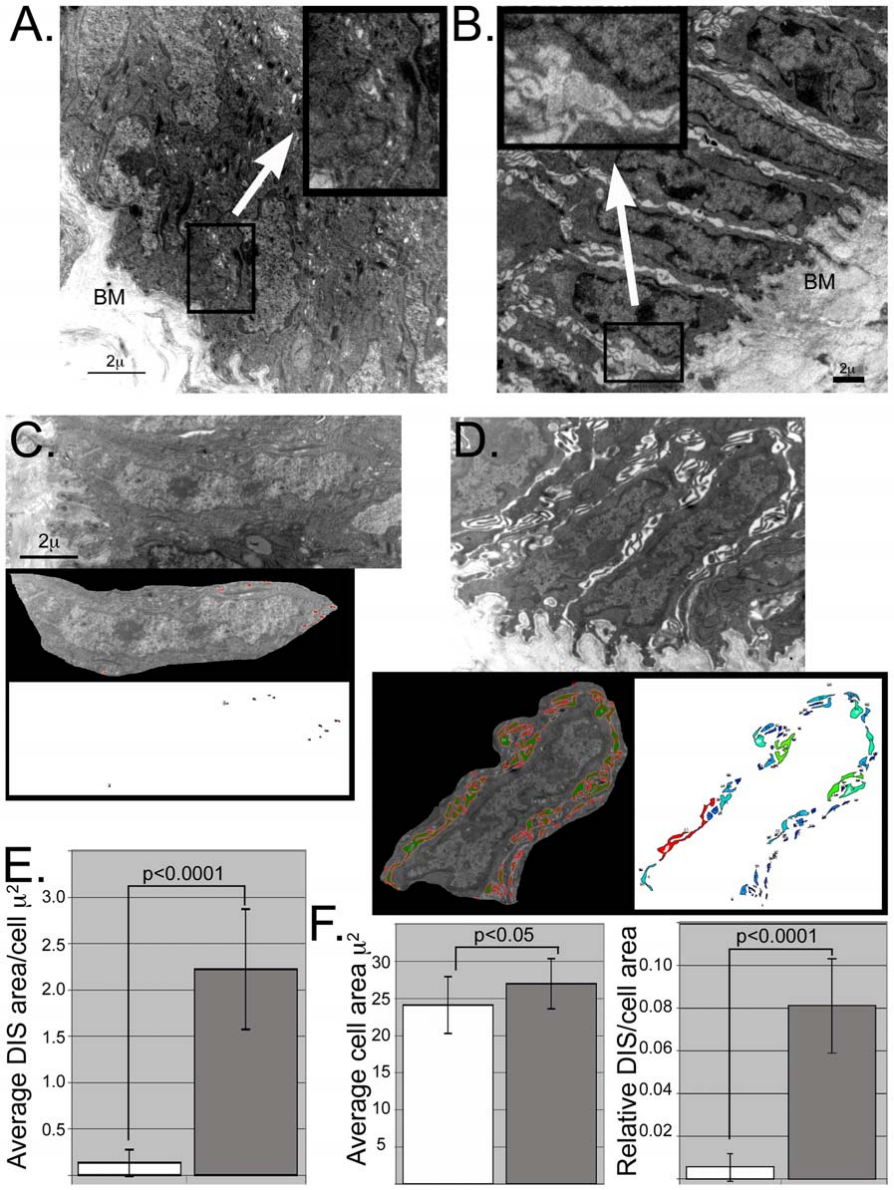
Cdx2 expression is associated with dilated intercellular space between basal cells and diminished barrier function. TEM images from **A.** wild-type and **B.** transgenic mice illustrating the increased space between basal keratinocytes. **Inset:** higher-powered image of intercellular space. **BM** = basement membrane. **C.** and **D.** Example of DIS mapping by IPLab software. Areas of low-density in the cut-out TEM image were identified, filled in with false color and quantified for wild-type (**C.**) and transgenic (**D.**) basal keratinocytes. **E.** Average intercellular empty space per cell, in microns^2^. Results are the combined measurements from two wild-type mice (5–6 cells/mouse, n = 11) and four transgenic mice (5–10 cells/mouse, n = 26). p values determined by Student's T test. **F.** Average of cell area for cells examined, and the ratio of intercellular area to cell area.

Cell-cell adhesive junctions appeared weakened. There were fewer desmosomes visible in the transgenic basal layer compared to controls. Moreover, there was more unfilled space between basal cells of the transgenic mouse (Figures 3C, 3D, 4A, and 4B). This increased intercellular space is reminiscent of the TEM phenomena Dilated Intercellular Spaces (DIS). DIS has been associated with acid reflux disease in humans and rabbit esophageal mucosa after experimental acid exposure [36], [37]. In addition, DIS has been associated with diminished barrier function in esophageal epithelium [38].

To quantify the DIS induced by Cdx2 expression, we used IPLab image processing software to map and measure areas of reduced density around cells. For this analysis, a single basal keratinocyte, including cell-cell junctions (but not cell-basement membrane junction) was cut from the original image, and regions of reduced density plotted and quantified by the software (Figures 4C and 4D). Using this technique, the significant difference in intercellular space between wild-type and transgenic basal cells is plainly seen. While wild-type basal cells had small regions of open space between cells, and minimal variability between individual basal cells, the *K14-Cdx2* basal cells had much more visible and variable areas of DIS. These areas were then quantified and averaged over multiple cells from several different animals. The average open space surrounding *K14-Cdx2* keratinocytes is 17-fold greater than that seen in wild-type cells (Figure 4E). Even after taking into account that the *K14-Cdx2* basal keratinocytes were, on average, slightly larger than wild-type cells (Figure 4F), the difference between transgenic and wild-type was still nearly 15-fold (Figure 4E). In summary, expression of Cdx2 in basal keratinocytes of the esophagus and forestomach alters cell morphology and leads to dilated intercellular spaces reminiscent of acid injury.

### Ectopic Cdx2 expression in basal keratinocytes reduces Desmocollin 3 levels and epithelial barrier function

**Figure 5:**
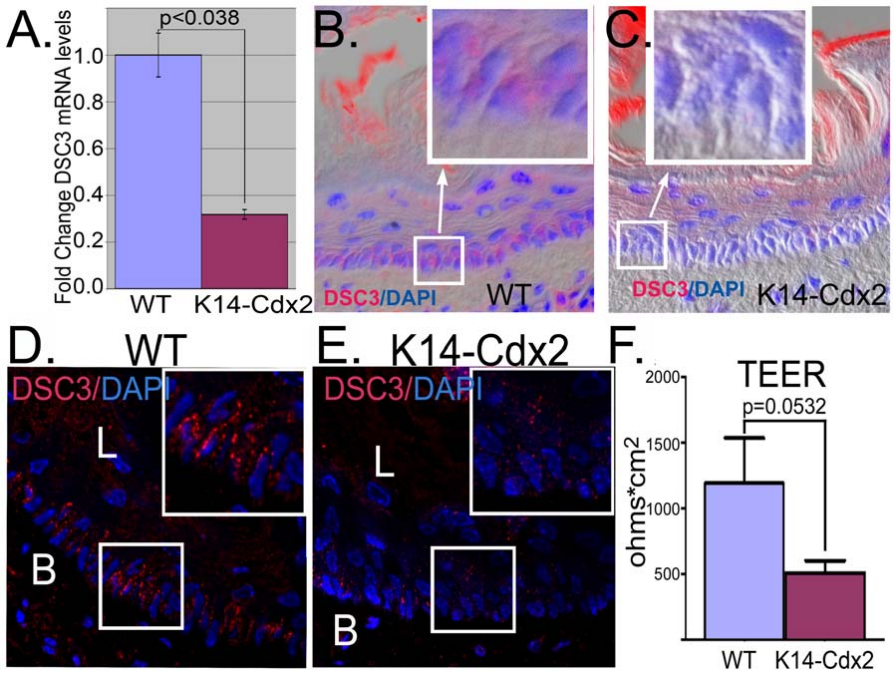
DSC3 levels are reduced in *K14-Cdx2* mice and are associated with diminished trans-epithelial electrical resistance. **A.** DSC3 mRNA levels quantified by QPCR in *K14-Cdx2* and wild-type (**WT**) mice. n = 3 mice for each measurement. **B.** Epifluorescent staining for DSC3 (**Red**) in basal cells in the esophageal epithelium from wild-type mouse. Nuclei counterstained with DAPI. The image is a merge of the fluorescent channels with a differential interference contrast (DIC) image of the tissue. **C.** Epiflourescent image of DSC3 (**Red**) in *K14-Cdx2* esophageal epithelium, counterstained with DAPI and merged with DIC. **D. and E.** Confocal examination of DSC3 (**Red**) protein in wild-type (**D**) and *K14-Cdx2* (**E**) esophageal epithelium. **F.** Mean TEER (±SEM) for wild-type (n = 18) and *K14-Cdx2* (n = 19) forestomach epithelium as determined using a mini-Ussing chamber. Measurements were log-transformmed to achieve approximate normality, then a two-sample t-test was performed to determine statistical significance of the difference.

We expected that the molecular processes contributing to the measurable increases in intercellular spaces might be due to alterations in cell-cell adhesion proteins. E-cadherin, Claudin-1 and Claudin-5 mRNA levels appeared unchanged by the transgene expression (Figure S2). Immunohistochemical staining for E-cadherin suggested some reduction in E-cadherin protein levels, but this reduction was patchy and did not appear to be the primary cause for the diminished cell-cell adhesion (Figure S2). We noticed there were fewer desmosomes in the basal layer cells on the TEM images (Figure 3CD and data not shown). Desmocollins (Dsc) are members of the cadherin superfamily of proteins and are critical components of desmosomes [39]. Dsc3 is typically expressed in multilayered squamous epithelium in the basal cell layer. Quantitative PCR analysis for the expression of Desmocollins 3 was performed. Dsc3 mRNA levels were significantly reduced, by about 75% compared to controls (Figure 5A). Histologically, we see evidence for a significant reduction in Dsc3 proteins levels as well. Dsc3 protein is localized to primarily the basal cell layer of the esophagus (Figure 5B). Levels of Dsc3 protein appear considerably reduced in the *K14-Cdx2* mice (Figure 5C). Since the Dsc3 staining was somewhat diffuse by epifluorescence, we reimaged the tissues by confocal microscopy. Dsc3 staining was punctate rather than diffuse, as would be expected for desmosomes. Moreover, we can again clearly see significant reductions in Dsc3 proteins levels in the *K14-Cdx2* mice compared to littermate controls (Figure 5D and E).

In order to determine if this Cdx2-associated reduction in Dsc3 levels, reduction in desmosome numbers, and increased in intercellular space at the basal cell layer had any significant functional implications for the epithelia, we measured trans-epithelial electrical resistance (TEER) using a mini Ussing chamber [38]. The mouse esophagus is small and did not yield tissue with enough area to cover the chamber's opening. We therefore made the measurements using the squamous forestomach which, as we have demonstrated, expresses the transgene (Figure S1). We observed that TEER was significantly reduced in the *K14-Cdx2* mice when compared to littermate controls, suggesting an impairment of epithelial barrier function (Figure 5F). *K14-Cdx2* mice electrical resistance in the forestomach was less than half of wild-type controls. Together these findings establish that expression of Cdx2 in squamous epithelium diminishes cell-cell adhesion in the basal cell layer and reduces the epithelial barrier function.

### Basal epithelial cells with secretory features are induced in the *K14-Cdx2* mice

**Figure 6:**
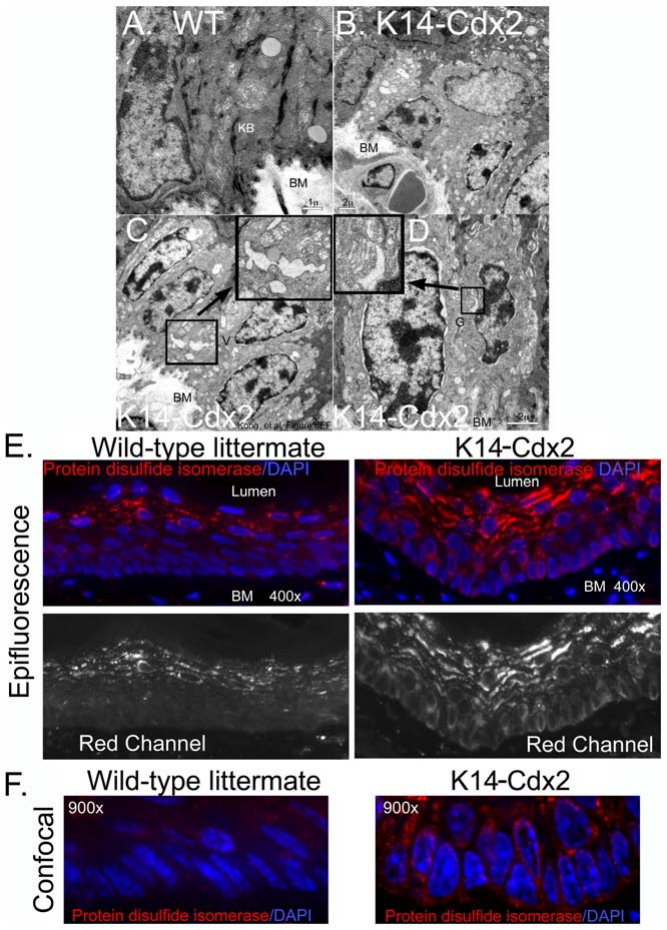
Transgene expression is associated with adoption of a more glandular ultrastructure. **A.** TEM of basal keratinocyte from wild-type mouse demonstrating keratin bundles in cytoplasm. **KB** = keratin bundle; **BM** = basement membrane. **B.** TEM of four “pale” basal cells in *K14-Cdx2* mice. **C.** TEM of another cluster of pale cells demonstrating increased numbers of cytoplasmic vesicles suggestive of a glandular or secretory cell ultrastructure. **Inset**: magnified vesicle. **D.** TEM of another set of secretory-type cells in the basal layer with prominent golgi. **Inset**: magnified golgi. **E. and F.** Levels and distribution of the endoplasmic reticulum marker protein disulfide isomerase are visualized with the **Red** **channel** by Epifluorescent (**E**) and confocal (**F**) microscopy. The tissue was counterstained with DAPI. **BM** =  basement membrane.

Ultrastructural analysis revealed another feature in the *K14-Cdx2* mice. A subpopulation of cells was observed that were different from the more traditional keratinocytes. These cells demonstrated a lower electron density (appeared pale) compared to adjacent keratinocyte cells. They were located along the basal cell layer, and they remained associated with adjacent basal epithelial cells through desmosomes, and with basal lamina through hemidemosomes (Figure 6A and B). These cells displayed a significant reduction of cytokeratin bundles and free ribosomes in the cytoplasm compared to adjacent keratinocytes, explaining their reduced electron density. More interestingly, they appeared to be developing extensive intracellular membrane compartment including Endoplasmic Reticulum (ER), Golgi apparatus, and other membrane vesicles (Figure 6C and D). These features are more consistent with glandular type epithelial cells with active secretory functions and are not characteristic of keratinocytes. They are reminiscent of the multilayered epithelium (MLE) ‘Distinctive’ cells based on this appearance [40], [41], [42].

To better establish whether these vesicles represented ER and/or golgi structures, we stained for protein disulfide isomerase (ER) and giantin (golgi). These proteins are specific for the ER and golgi, respectively [43], [44]. Giantin levels appeared to be unchanged with Cdx2 expression in the esophagus (Figure S3). However, protein disulfide isomerase levels appeared considerably increased in the basal cell layer of the *K14-Cdx2* mice (Figure 6E and F). The staining pattern is heterogeneous, consistent with the TEM observation of heterogeneity as well. Little protein disulfide isomerase is detected in wild-type basal cells. Upper, differentiated layers appear to stain as well as the transgenic mice, but it is not clear if this is due to non-specific interactions of the antibody with the keratinizing cells. By confocal microscopy we observe clear perinuclear staining in the K14-Ccdx2 basal keratinocytes, as would be expected for an ER protein (Figure 6F). Together these findings suggest Cdx2 expression is associated with significant alterations in basal keratinocyte ultrastructure and cell identity.

### Cdx2 synergizes with DNA methyltransferase inhibitor to induce Barrett's esophagus and intestinal genes

While ectopic expression of Cdx2 had several significant effects on keratinocyte cell biology and appears to have induced a cell lineage with increased secretory ultrastructure, there was no significant induction of intestinal or Cdx2-target genes. Alcian blue staining for mucin-producing cells was negative, as was qPCR and immunohistochemical studies for Muc-2, Sucrase isomaltase, Lactase phlorzin hydrolase, Carbonic anhydrase I, alkaline phosphatase, Down-regulated in adenoma (S26A3/DRA), Na-H Exchanger 2 (NHE2), and Guanyl Cyclase-C (data not shown).

**Figure 7:**
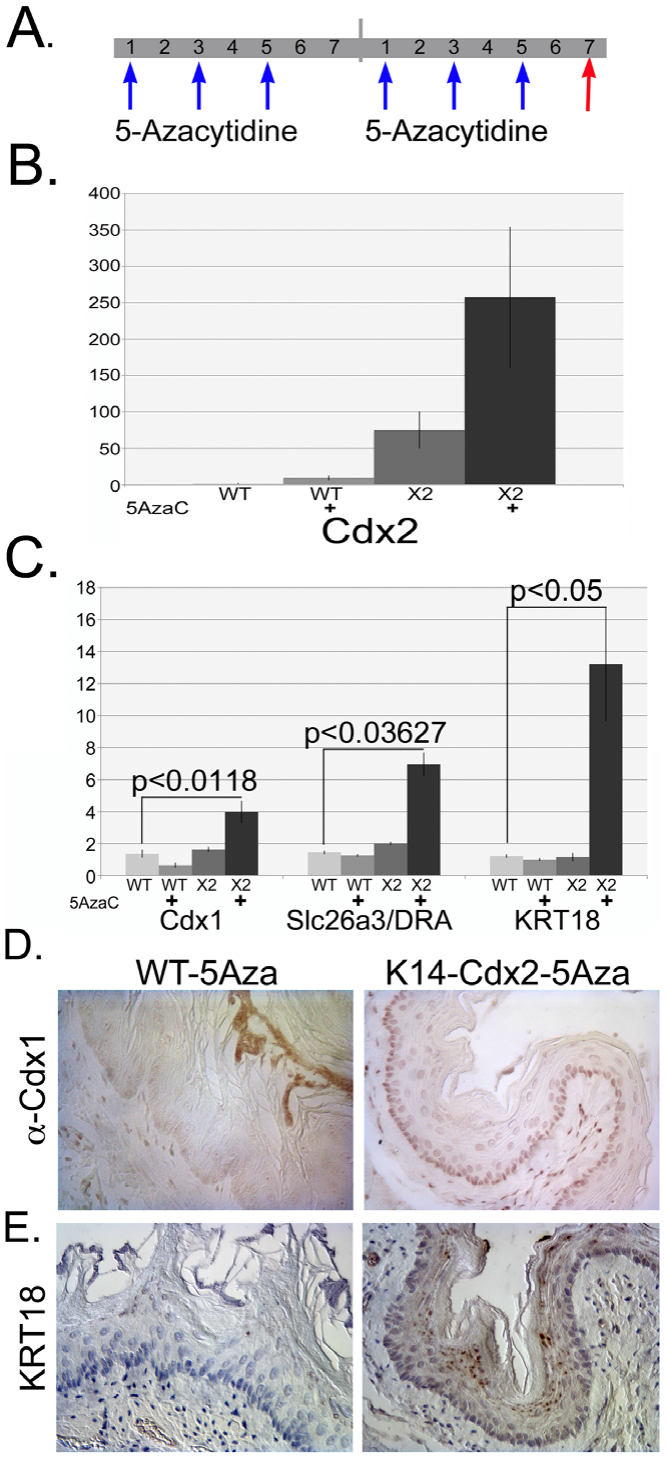
Treatment of *K14-Cdx2* mice with DNA methyltransferase inhibitors induces Cdx2 targets and BE-associated genes. **A.** 1mg/kg of 5′-Azacytidine was injected intraperitoneally on the indicated days (**Blue arrow**) and the mice were sacrificed on day 14 (**Red arrow**), 2 days after the last injection. **B.** qPCR measurements of Cdx2 mRNA levels, n = 6. **WT** = wild-type mice; **X2** = K14-Cdx2 mice. C. Determination of Cdx1, Slc26a3/DRA, and KRT18 mRNA levels by qPCR, n = 6. **D.** Cdx1 protein by immunohistochemistry in treated wild-type and *K14-Cdx2* mice. **E.** Keratin-18 by immunohistochemical staining.

Previously, we observed that Cdx2-expression in a human esophageal keratinocyte cell line similarly failed to induce known Cdx2 gene targets [11]. We found, however, that treatment of these cells with 5′-Azacytidine (5Aza), a DNA methyltransferase inhibitor, lead to a Cdx2-specific induction of a number of Barrett's associated genes including SLC26A3/DRA, NHE2, and Keratin-20. This suggested epigenetic mechanisms were involved in the inhibition of Cdx2 gene targets in human esophageal keratinocytes. To determine if the same process was silencing Cdx2 target genes in the *K14-Cdx2* mice, we treated the mice with intraperitoneal (IP) injections of 5Aza (1mg/kg) three times a week for two weeks (Figure 7A). The mice were then sacrificed, and portions of their esophagi were fixed and embedded for histology, and the remainder used for qPCR analysis. Histologic evaluation of the esophagi from treated *K14-Cdx2* and control mice revealed no obvious changes in tissue morphology (data not shown).

However, qPCR studies revealed several significant changes. Cdx2 mRNA levels were increased by 10-fold in wild-type mice, and 250-fold in *K14-Cdx2* mice treated with 5Aza (Figure 7B). Of greater interest, mRNA levels from the Cdx2 homologue, Cdx1, were significantly increased, but only in treated *K14-Cdx2* mice (Figure 7C). Similarly, SLC26A3/DRA and Keratin-18 (Krt18) levels were significantly increased, but only in *K14-Cdx2* mice treated with 5Aza (Figure 7C). All three genes are known to be ectopically expressed in Barrett's esophagus along with Cdx2, and both Cdx1 and SLC26A3/DRA are known Cdx2 gene targets [10], [11]. The increased mRNA expression resulted in detectable Cdx1 and Krt18 protein by immunohistochemistry in the *K14-Cdx2* mice treated with 5Aza but not similarly treated wild-type mice (Figure 7D and E). Several other genes, including p16, CAI, Krt20, and Muc2 were not noticeably induced by 5Aza treatment in the *K14-Cdx2* or control mice (data not shown). We conclude that *K14-Cdx2* mice can be induced to express more Cdx2 gene targets and BE-associated gene markers if chromatin remodeling and/or epigenetic DNA changes can be elicited.

## Discussion

Barrett's esophagus is the replacement of squamous epithelium in the esophagus with a columnar epithelium expressing intestinal features. BE itself is asymptomatic [1]. However, BE has also been identified as a predisposing lesion for the development of esophageal adenocarcinoma [1], [2], and therefore remains a clinically important condition. Our studies elucidate the consequences of ectopic Cdx2 expression in esophageal epithelium, and models an intermediate stage in the emergence of an intestinalized epithelium from normal squamous epithelium.

### Ectopic Cdx2 expression in the esophagus is associated with significant changes in epithelial function

Expression of Cdx2 mRNA has been detected in esophageal epithelium in gastroesophageal reflux patients in the absence of BE [30], in the normal appearing squamous epithelium proximal to established BE [45], and can be provoked in esophageal keratinocytes in vitro when cultured under conditions of acidic or bile acid stress [6], [31]. The level of Cdx2 protein expressed within these cells can vary. Nuclear-localized Cdx2 protein is observed in columnar epithelium of BE and in the *in vitro* cultures of acid-treated keratinocytes. There also appears to be a low-level of Cdx2 protein localized in the cytoplasm of squamous epithelial cells from GERD patients[30], [46], [47]. However the consequences of this ectopic expression for epithelial integrity and cell biology have not been explored.

Our findings in the *K14-Cdx2* mouse establishes that this expression is associated with two significant effects, reduced cell proliferation, and diminished barrier function. The reduction in cell proliferation is significant and correlates with our previously published study using primary immortalized human esophageal keratinocytes [11]. While this reduction was not associated with any deleterious effects in the normal mouse, we anticipate that under stress conditions, such as in the setting of GERD induced injury, the ability of the epithelium to undergo repair and replace dead cells may be significantly impacted. Studies are underway to stress the epithelium of *K14-Cdx2* mice by physiologic processes that mimic reflux. We anticipate observing significant differences when this epithelium is stressed compared to wild-type mice.

Moreover, the significantly dilated intercellular spaces (DIS) we noted in the basal cell layer of the *K14-Cdx2* mice are associated with increased paracellular permeability in the esophagus and forestomach, as demonstrated by the significantly reduced TEER. This is consistent with published reports in which acid-provoked DIS has been associated with diminished barrier function [38]. While the ultrastructure appearance resembles DIS associated with acute acid injury in animal models [36], we do not suspect acid is the cause of this DIS. Rather, the Cdx2 mediated reduction cell-cell adhesion appears to be the critical event. E-cadherin protein levels are somewhat disturbed, however E-cadherin, Claudin-1, and Claudin-5 mRNA levels are entirely unaffected. In contrast, Dsc3 mRNA and protein levels are both significantly diminished, suggesting this may be the primary event, with effects upon E-cadherin and other cell adhesion processes affected secondarily. Disruption of desmosome formation may make all cell-cell adhesive junctions less stable, leading to increased E-cadherin turn-over and diminished protein levels. Future studies will examine the molecular events leading to diminished Dsc3 gene expression in *K14-Cdx2* mice. We conclude that ectopic cdx2 expression in basal esophageal keratinocytes significantly impacts critical epithelial functions including cell proliferation and barrier permeability.

### A subpopulation of *K14-Cdx2* esophageal epithelial cells resembles a transitional cell type associated with Barrett's esophagus

The emergence of the specialized columnar epithelium characteristic of BE within a stratified squamous epithelium has been difficult to explain. Indeed, the identity of the progenitor cell that gives rise to Barrett's epithelium remains an open question [48], [49]. It is expected that the progression to BE is a stepwise process, with intermediate stages in between. While no BE precursor lesion is widely accepted, there has been emerging data regarding an altered epithelium known as multilayered epithelium (MLE) [50], [51], [52], [53]. MLE is described as a hybrid epithelium, with squamous cells in the basal portion and columnar cells layered above, and is thought to represent a transitional stage or intermediate cell type in the squamous to columnar conversion to metaplasia in BE. Gene expression patterns are also hybrid in this cell type, with both squamous cytokeratins (*KRT4* and *KRT13*) and columnar (*KRT8* and *KRT19*) detected in the MLE.

At the ultrastructural level, MLE is marked by the presence of a ‘Distinctive cell’, characterized by microvilli (columnar cell feature) and intercellular ridges (squamous cell feature). Distinctive cells also possess abundant secretory-type intracellular vesicles (columnar cell feature) [40], [41], [42]. Pathologically, MLE is usually detected at or near the neo-squamocolumnar junction in BE, and is strongly correlated with reflux. Rat surgical models of acid and bile reflux similarly induce MLE [54], [55], [56]. Studies have found Cdx2 expression in both human MLE [55] and the surgically-provoked MLE in rats [56]. Based on these findings, ectopic Cdx2 expression may contribute to the development of MLE, a transitional cell type between squamous and columnar cells in BE.

Thus, the ultrastructural findings of pale cells with abundant cytoplasmic endoplasmic reticulum in the *K14-Cdx2* mice were both very novel and exciting. These cells resemble a transitional cell type between keratinocytes and columnar secretory cells. Moreover, they share many features of the ‘Distinctive cell’, except the *K14-Cdx2* ‘Distinctive cells’ are located in the basal cell compartment and do not have visible microvilli. These findings are the first experimental evidence to demonstrate that ectopic Cdx2 expression in keratinocytes generates cells with features of the ‘Distinctive cell in MLE’. It is interesting to speculate whether the increased ER levels observed in these ‘Distinctive’ cells provides them a protective advantage by fostering quicker cell membrane repairs after acid and bile injury, or better control of intracellular pH when stressed by GERD. Alternatively, with the reduced cell proliferation, cell-cell adhesion, and barrier function, this intermediate cell stage may be unstable in the setting of active reflux and likely to progress to a more complete intestinal morphology and ultrastructure. This will be a focus for future studies.

The absence of a mature BE columnar cell morphology in our transgenic mice was not unexpected. Despite Cdx2's required role in intestinal epithelial development [20], as a single factor it does not appear able to reprogram a cell once a developmental program has been initiated. Timing of the expression appears to be critical. Cdx2 disruption early in development leads to failure of intestinal epithelial development and its replacement with a multi-layered squamous epithelium due to an anterior homeotic identity shift [20]. However, loss of Cdx2 after adoption of an intestinal identity causes a disruption in cell differentiation and polarization, but not the adoption of a squamous cell identity [57]. The K14 promoter is active in the esophagus early during development but after the cells have adopted a keratinocyte identity[58]. We selected this promoter, rather than one active earlier in development, because we were trying to model the transition to Barrett's esophagus in adults rather than simply modulate differentiation programs during development. The induction of the endoplasmic reticulum-laden cells is a very strong indication that the transition to a columnar-type metaplasia from a keratinocyte is a possibility if the correct gene combinations and cellular microenvironment can be induced.

One reason that developmental patterns are difficult to disrupt once established is that chromatin remodeling usually occurs to silence competing regions of the genome [59], [60]. Therefore, ectopic expression of critical transcription factors may not initiate new developmental patterns unless chromatin remodeling occurs. Moreover, there is evidence of significant differences in epigenetic gene regulation between normal squamous epithelium and Barrett's esophagus [61], [62], [63], [64]. Our use of 5-Aza was an attempt to open these areas of silenced chromosomes to Cdx2 transcriptional activity. Our induction of Cdx2 and Keratin18 protein in the *K14-Cdx2* mice by treatment with 5′-Azacytidine supports this hypothesis and serves as the basis for future studies. However, 5′-Azacytidine non-specifically demethylates DNA by inhibiting DNA methyltransferase activity. This broad effect, in the absence of any selective pressure (such as from acid and bile reflux), may be why we observe some gene expression changes but no significant change in epithelial cell morphology. Future experiments will try to induce a GERD environment to provide selective pressure for the emergence of a more Barrett's like epithelium.

In summary, we establish the *K14-Cdx2* mouse as a useful animal model for studies of BE pathogenesis. Ectopic Cdx2 expression in the esophagus can reduce epithelial cell proliferation and diminish the epithelial barrier function while inducing a subpopulation of cells with diminished keratin bundles and increased endoplasmic reticulum. Thus our findings suggest the expression of a single transcription factor, Cdx2, is sufficient to induce a population of cells in transition between squamous keratinocytes and columnar BE cells. We anticipate these mice will be useful in studies of BE pathogenesis as we employ a variety of strategies to advance the metaplasia phenotype, and test whether these transitional cells have acquired any advantage to respond to GERD conditions. These are important avenues we plan to explore in future studies.

## Materials and Methods

### Generation of K14-Cdx2 transgenic mice

All studies with the mouse models was fully approved by the Institutional Animal Care and Use Committee (IACUC) at the University of Pennsylvania (IACUC#525400). The University of Pennsylvania is fully accredited by the American Association for Accreditation of Laboratory Animal Care, and the animal care and use program conforms to all required standards. A cDNA comprising the coding region of murine Cdx2 (1035 bp) was inserted into the Bam HI site of the K14/hGH expression vector (a kind gift of Elaine Fuchs, The Rockefeller University, New York, New York) [33]. This Keratin 14 (K14) promoter is active in the basal layer of the squamous epithelium for the esophagus, tongue, oral mucosa, and skin. The resulting plasmid was digested with EcoRI and used for microinjection of fertilized ova of FVB/N mice (Taconic, Germantown, NY). Transgenic founders were identified by polymerase chain reaction (PCR) and confirmed by southern blot analysis and bred to establish lines. All PCR primers listed in Table S1. Transgenic founders of the BGSJL/F1 strain (Jackson Laboratory, Bar Harbor, ME) were bred with normal CD-1 mice and backcrossed to C57BL/6 (Charles River, Wilmington, MA) for 3 generations. All animals were bred under pathogen-free conditions and used for experiments at 6–12 wk of age. All experiments with mice were conducted with the approval and in compliance with the guidelines of The Institutional Animal Care and Use Committee (IACUC) of the University of Pennsylvania School of Medicine.

### Isolation and culturing of primary mouse esophageal keratinocytes

Esophagi were isolated from young mice (1–3 months), opened longitudinally, washed in PBS, and incubated with 0.6–1.0 U/ml of Dispase 1 (Roche Applied Science, Mannheim, Germany) for 15 minutes at 37°C, and epithelial sheets were stripped from mesenchymal tissues using forceps [65]. The epithelial sheets were minced and incubated with 0.05% trypsin/EDTA (Gibco) for 10 minutes at 37°C. The trypsin was then quenched with 200 µg/ml soybean trypsin inhibitor (Sigma-Aldrich, St Louis, MO) and the suspension passed through a 40-µm cell strainer (Becton Dickinson). Cells were centrifuged and resuspended in keratinocyte serum-free medium (KSFM; Gibco) with EGF (1 ng/ml), BPE (50 mg/ml).

### Western Blot Analysis

Total proteins were isolated from the esophageal epithelium of 3-month old mice by an adaptation of a previously described method [66]. Frozen esophageal tissue was thawed and homogenized using a Potter-Elvehjem homogenizer at 4°C in the presence of Complete Protease Inhibitor Cocktail (Boehringer Mannheim, Indianapolis, IN). Protein concentration was determined by the BCA protein assay (Pierce, Rockford, IL). Western blot analysis was then conducted. The blot was incubated with monoclonal Cdx2 antibody (clone CDX2-88) (1:1000; BioGenex, San Ramon, CA), and then visualized with secondary anti-mouse horseradish peroxidase-conjugated antibodies and chemiluminescence detection (ECL; Milipore). To verify equal loading of samples, the blots were stripped and reprobed with anti-tublin (Sigma, St Louis, MO) at 1∶1500.

### Quantitative Reverse Transcription-Polymerase Chain Reaction

Samples were stored in tissue storage reagent (RNAlater; Ambion, Austin, TX). Total RNA was isolated using RNeasy Mini kit (Qiagen, Valencia, CA). cDNA was prepared from total RNA using the SuperScript® VILO™ cDNA Synthesis Kit (Invitrogen, Carlsbad, CA). Primers were designed using Primer Express software (Applied Biosystems). All PCR primers are listed in Table S1. Taqman primers for Desmocollin 3, villin, DRA, NHE2, which were obtained from Applied Biosystems (Foster City, CA). Quantitative RT-PCR was performed on an ABI 7000 sequence detection system (Applied Biosystems, Foster City, CA), with SYBR green or Taqman as the fluorescent dye using standard PCR conditions. A dissociation curve was run with each PCR as a control. A ribosomal phosphoprotein, 36B4, was used as the normalization control. ΔC^t^ values were calculated after duplicate PCRs for each sample as described, [22], [67] then statistical analysis performed. ΔΔC^t^ values were then calculated and used to determine fold-change in expression.

### Transepithelial electrical resistance (TEER)

Mouse forestomachs were excised and placed in buffer containing in mM: 120 NaCl, 25 NaHCO_3_, 3.3 KH_2_PO_4_, 0.8 K_2_HPO_4_, 1.2 MgCl_2_, 1.2 CaCl_2_, and 10 glucose. Forestomachs were individually pinned across a 0.3 cm^2^ opening in a specialized slider (P2304, Physiologic Instruments, San Diego, CA) for insertion into a vertical Ussing chamber (Physiologic Instruments). Mucosal and serosal baths were the same buffer in which tissue was stored following excision. While in the Ussing chamber, tissues were kept at 37°C and the solutions were constantly mixed by gas lift with 95% O_2_/5% CO_2_ such that solution pH was kept between 7.35 and 7.45. Tissues were voltage-clamped continuously to 0mV after fluid resistance compensation using an automatic voltage clamp (VCC 600, Physiologic Instruments). Short-circuit current (I_SC_) was digitized at 1 sample every 10 seconds, and data were stored on a computer hard drive using Acquire and Analyze software build 2.3.0 (Physiologic Instruments). Transepithelial resistance (R_T_) was determined automatically by the software using a protocol for stepping the clamp potential from 0 mV to +/− 10 mV for 240 ms every minute, recording the change in I_SC_, and calculating R_T_ from Ohm's law (R_T_  =  DV/DI). The average R_T_ over 2 m was taken as the tissue resistance.

### Immunohistochemical and Immunofluorescence analyses

All specimens were isolated, rinsed in ice-cold PBS, fixed, and analyzed histologically by staining sections with hematoxylin and eosin (H&E) or immunohistochemically using standard methods as described [68]. Five-µm paraffin-embedded sections were pretreated with xylene and then placed in a microwave oven in 10 mmol/L citric acid buffer. Endogenous peroxidases were quenched using hydrogen peroxide before sections were incubated in avidin D blocking reagent and biotin blocking reagent. Primary antibodies used include monoclonal Cdx2 (1∶200, Biogenex, San Ramon, CA), p63 (1∶500, gift of Makoto Senoo, University of Pennsylvania, Philadelphia, PA), Cdx1 CPSP (1∶750) [35], ER marker PDI (1∶1,000, Cell Signaling Technology #3501, Beverly, MA) and for proliferation using bromodeoxyuridine (BrdU) antibody (1∶1,500, Upstate, Charlottesville, VA). Sections were incubated with primary and biotinylated secondary antibodies and an avidin-horseradish peroxidase conjugate (Vectastain Elite ABC kit; Vector Laboratories, Burlingame, CA) following the manufacturer's protocol. The signal was developed using the 3,3′-diaminobenzidine substrate kit (Vector Laboratories). Sections were counterstained with hematoxylin. The serial sections were stained for Cdx2 and p63 by immunofluorescence using antibodies listed in Table S2.

For Alcian blue staining, slides were deparaffinized. After application of 3% aqueous acetic acid to the slides, 1% Alcian blue in 3% acetic acid, pH 2.5, was applied. Sections were washed and counterstained with 0.1% nuclear fast red, dehydrated, and mounted. For immunofluorescence detection of Golgi marker on the esophageal reconstructs, frozen tissue sections were fixed with 4% Paraformaldehyde for 3-5 minutes and blocked with Protein Blocking Agent (StartingBlockTM T20Blocking Buffer Thermo Scientific #37539) at room temperature for 10 minutes. Primary anti-Giantin (ab24586, Abcam, Cambridge, MA, US) antibody at a concentration of 1∶1,000 were incubated overnight at 4°C. Slides were washed with PBS four times, and secondary anti-rabbit antibody was incubated at 37°C for 30 minutes. After incubation, slides were washed with PBS four times, counterstained with DAPI, and then photographed under a Nikon E600 fluorescent microscope and confocal microscope.

### BrdU labeling/Proliferation Studies

BrdU incorporation rate was measured in the basal layer of transgenic and non-transgenic littermate controls. Mice were injected with BrdU (Zymed) at 1.0 mg/ml 1 hour before sacrificing. Mice were sacrificed and their esophagi were collected and fixed in 4% PFA. Esophageal tissues from WT mice were used as controls. After BrdU immunohistochemistry, BrdU-labeled nuclei were counted in the basal layer from regions of the esophagus. Positive cells were expressed as the number of BrdU(+) cells per crypt unit. Three transgenic and matched nontransgenic littermates control mice from each of the three *K14-Cdx2* lines were measured in this study. Ten random visual fields were analyzed per esophageal section, and percent BrdU–positive cells was determined using IPLab Software (Scanalitics, Fairfax, VA). At least 500 cells were measured in each mouse. IP Lab was used to quantitate cell staining of BrdU.

### Transmission Electron Microscopy (TEM) ultra-structural analysis

For TEM, tissues were fixed and analyzed as described previously described [22]. For transmission electron microscopy (TEM), tissues were fixed in 2.5% (w/v) glutaraldehyde and 2.0% paraformaldehyde in 0.1 M cacodylate buffer (pH 7.4) at 4°C overnight. After buffer wash, the samples were post-fixed in 2% osmium tetroxide for 1 h, washed again in buffer, and dehydrated in a graded ethanol series. Samples were treated with several changes of hexamethyldisilazane (HMDS) and then allowed to air dry prior to mounting and sputter-coating with gold. TEM examinations were made, and three to five TEM photos/per animal were taken with a Philips XL20 TEM microscope.

The quantitation of intercellular space between basal layer cells of the esophageal epithelium was measured using IPLab software (Scanalytics, Fairfax, VA). Intercellular spaces were delineated in between 5–10 epithelial cells from the basal layer of each mouse. Two wild-type and four transgenic mice were used in these studies. The region to be analyzed was outlined manually. It included the cell and the cell membranes from all adjacent cells, but not the bottom of the cell where it attaches to the basement membrane. The software then identified low-density pixels, outlined contiguous ones, and computed their total area. Then the cell area not including the DIS was outlined manually and the total cell area computed. For statistical analysis, all cell data were combined and DIS, cell area, and DIS/cell area averaged across control and transgenic mice.

### Statistics

All data is expressed as mean ± Standard deviation unless indicated. Single comparisons were performed by Student's t-test when appropriate. The distribution of trans-epithelial electrical resistance (TEER) in both the wild-type and K14-Cdx2 mice groups was right-skewed. Hence a log-transformation was used to achieve approximate normality (Shapiro Wilk test for normality p-value = 0.8557 for K14-Cdx2 and 0.5098 for wild-type mice). A two-sample t-test on the log transformed resistance measurements was performed to determine statistical significance of the difference (t = 2.00, df = 35, p-value = 0.0532).

## Supporting Information

Figure S1 **Cdx2 expression in** ***K14-Cdx2*** **forestomach and TUNEL assays.** Immunohistochemistry for **A.** Cdx2 in squamous forestomach from *K14-Cdx2* mice. **B.** Anti-mouse secondary Ab staining controls for Cdx2 immunohistchemistry. **C.** TUNEL staining for apoptotic cells in Wild-type littermate esophagus. **D.** TUNEL staining for apoptotic cells in *K14-Cdx2* mouse esophagus.
(TIF)

Figure S2 **Quantitative PCR for cell-cell adhesion proteins in** ***K14-Cdx2*** **mice.** **A.** mRNA levels for E-cadherin by qPCR in 3 month old *K14-Cdx2* and control littermate mice; n = 5; p values determined by Student's T test. Immunohistochemistry for E-cadherin in the esophagi from 3 month old **B.** control littermate mice or **C.** *K14-Cdx2* mice. mRNA levels for **D.** Claudin-1, and **E.** Claudin-5 by qPCR.
(TIF)

Figure S3 **Levels and distribution of the golgi protein Giantin are unchanged by the Cdx2 transgene.** The levels and distribution of the golgi protein Giantin **(Red)** are visualized by epifluorescent microscopy in esophageal epithelium from **A.** Wild-type (WT) and **B.** *K14-Cdx2* transgenic mice. Nuclei were counterstained with DAPI (**Blue**). **BM** =  basement membrane. Higher power, confocal microscopy evaluation for Giantin levels and distribution in **C.** Wild-type and **B.** *K14-Cdx2* mice.
(TIF)

Table S1 **Antibodies used in this study.**
(DOC)

Table S2 **qPCR Primers used in this study.**
(DOC)
