# Supplementary figures and images for: Ectopic Cdx2 Expression in Murine Esophagus Models an Intermediate Stage in the Emergence of Barrett's Esophagus
Source: PLoS One. 2011 Apr 6;6(4):e18280. doi: 10.1371/journal.pone.0018280 (PMC3071814; doi:10.1371/journal.pone.0018280)

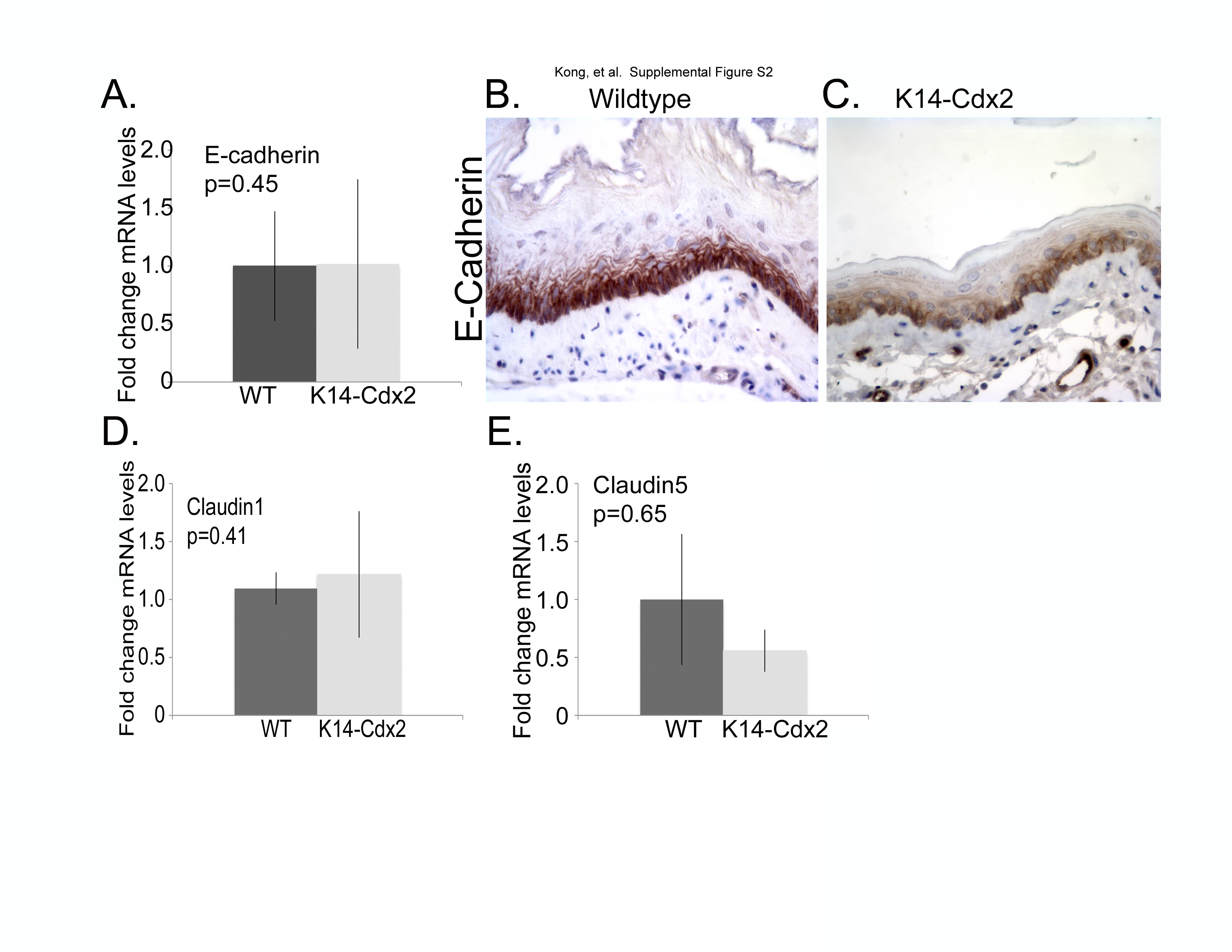

Supplement: Figure S2 — Quantitative PCR for cell-cell adhesion proteins in K14-Cdx2 mice. A. mRNA levels for E-cadherin by qPCR in 3 month old K14-Cdx2 and control littermate mice; n = 5; p values determined by Student's T test. Immunohistochemistry for E-cadherin in the esophagi from 3 month old B. control littermate mice or C. K14-Cdx2 mice. mRNA levels for D. Claudin-1, and E. Claudin-5 by qPCR. (TIF) [file pone.0018280.s002.tif]

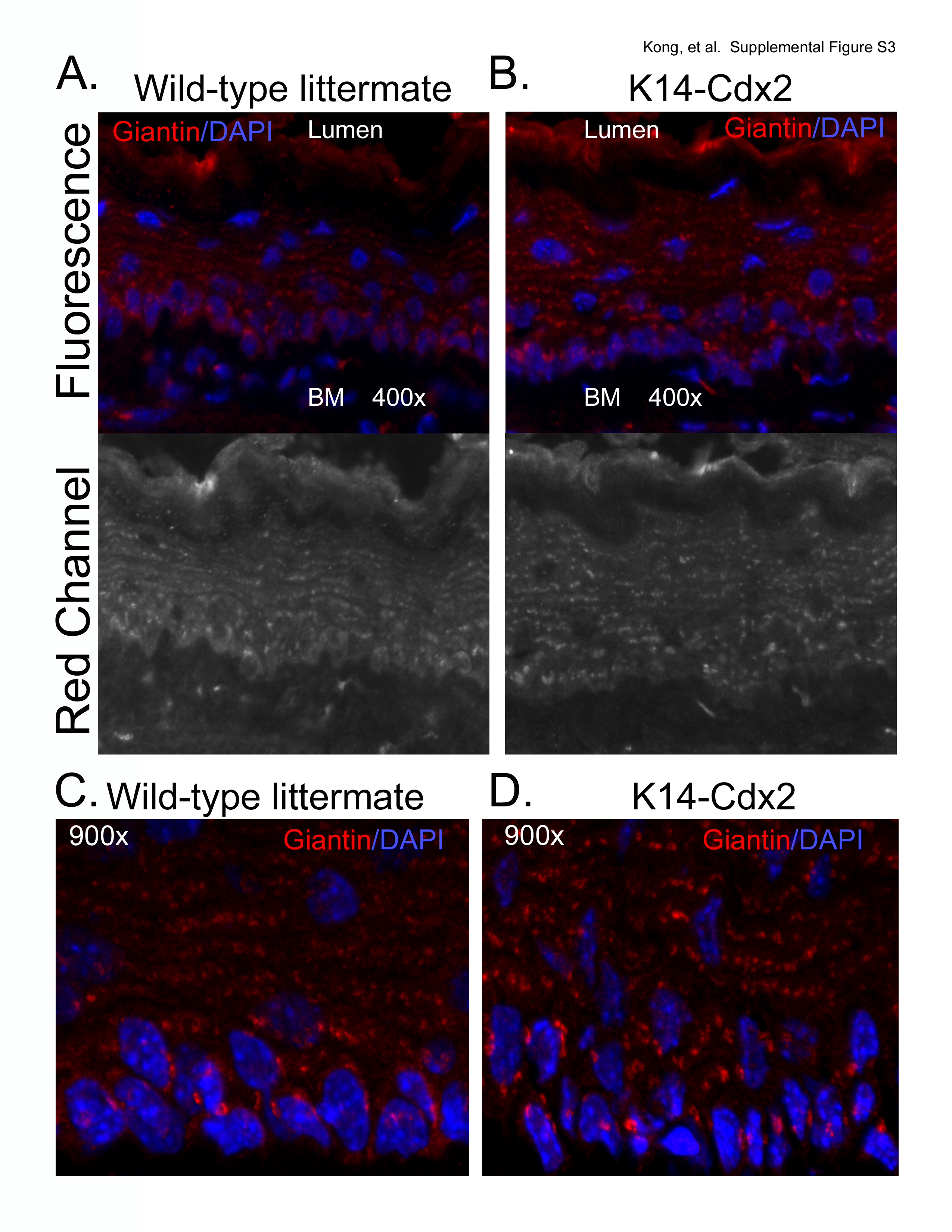

Supplement: Figure S3 — Levels and distribution of the golgi protein Giantin are unchanged by the Cdx2 transgene. The levels and distribution of the golgi protein Giantin (Red) are visualized by epifluorescent microscopy in esophageal epithelium from A. Wild-type (WT) and B. K14-Cdx2 transgenic mice. Nuclei were counterstained with DAPI (Blue). BM = basement membrane. Higher power, confocal microscopy evaluation for Giantin levels and distribution in C. Wild-type and B. K14-Cdx2 mice. (TIF) [file pone.0018280.s003.tif]
