# Supplementary material for: Ectopic Cdx2 Expression in Murine Esophagus Models an Intermediate Stage in the Emergence of Barrett's Esophagus
Source: PLoS One. 2011 Apr 6;6(4):e18280. doi: 10.1371/journal.pone.0018280 (PMC3071814; doi:10.1371/journal.pone.0018280)
Supplement: Table S1 — Antibodies used in this study. (DOC) [file pone.0018280.s004.doc]

TABLE S1 Antibodies used in this study

| Cdx2 | rabbit polyclonal Ab | Previously described |
| --- | --- | --- |
| Cdx2 | Mouse monoclonal | Biogenex |
| Cdx1 | rabbit polyclonal Ab | Previously described |
| keratin18 | mAb DC10 | cell signaling |
| ERp19 | Rabbit pcAb | Abcam |
| Golgi marker | ab24586 Rabbit pcAb | Abcam |
| p63 | mcAb | Abcam |
| BrdU ab | sheep pcAb | US Biological, Swampscott, MA |
