# Supplementary material for: Ectopic Cdx2 Expression in Murine Esophagus Models an Intermediate Stage in the Emergence of Barrett's Esophagus
Source: PLoS One. 2011 Apr 6;6(4):e18280. doi: 10.1371/journal.pone.0018280 (PMC3071814; doi:10.1371/journal.pone.0018280)
Supplement: Table S2 — qPCR Primers used in this study. (DOC) [file pone.0018280.s005.doc]

TABLE S2 qPCR Primers used in this study

| mCdx1 | F | 5'- AGACCGAACCAAGGACAAG-3 |
| --- | --- | --- |
|  | R | 5'-TGATGTACCGGCTGTAGTGAA-3' |
| mCdx2 | F | 5'-CCAAGTGAAAACCAGGACAAAA-3' |
|  | R | 5'-AACGAAGAAGCCCCAGGAA-3' |
| mKRT18 | F | 5'-CAAGTCTGCCGAAATCAGGGAC-3' |
|  | R | 5'-TCCAAGTTGATGTTCTGGTTTT-3' |
| mMuc2 | F | 5'-GCTGACGAGTGGTTGGTGAATG-3' |
|  | R | 5'-GATGAGGTGGCAGACAGAGAC-3' |
| mKRT20 | F | 5'-CCCAGAAGAACCTGCAAGAG-3' |
|  | R | 5'-ACGAGCCTTGACGTCCTCTA-3' |
| mKRT8 | F | 5'-GGACATCGAGATCACCACCT-3' |
|  | R | 5'-TGAAGCCAGGGCTAGTGAGT-3' |
| mKRT19 | F | 5'-CGGTGGAAGTTTTAGTGGGA-3' |
|  | R | 5'-AGTAGGAGGCGAGACGATCA-3' |
| mCA1 | F | 5'-TCACTGGGGCAACTCAAAC-3' |
|  | R | 5'-GCAGAATTCCAGTGAACTAAGTGA-3' |
| mLactase | F | 5'-CGTCTGCTTCCTATCAGGTTGAA-3' |
|  | R | 5'-GTGGGAAAATGTGTCCCAGATACT-3' |
| mPepT1 | F | 5'-CGTGCACGTAGCACTGTCCAT-3' |
|  | R | 5'-GGCTTGATTCCTCCTGTACCA-3' |
| Math1 | F | 5'-TCCCCTTCCTCCTACCTTCT-3' |
|  | R | 5'-CGATGCCACGTAAAGGTACA-3' |
| mTFF3 | F | 5'-CCTGGTTGCTGGGTCCTCTG-3' |
|  | R | 5'-GCCACGGTTGTTACACTGCTC-3' |
| mKRT14 | F | 5'-CAGCCCCTACTTCAAGACCA-3' |
|  | R | 5'-GGCTCTCAATCTGCATCTCC-3' |
| mBMP4 | F | 5'-GAGTTTCCATCACGAAGAACA-3' |
|  | R | 5'-GCTCACATCGAAAGTTTCCC-3' |
| mP16Ink4a | F | 5'-GTGTGCATGACGTGCGGG-3' |
|  | R | 5'-GCAGTTCGAATCTGCACCGTAG-3' |
| mP19Arf | F | 5'-GCTCTGGCTTTCGTGAACATG-3' |
|  | R | 5'-TCGAATCTGCACCGTAGTTGAG-3' |
| Notch1 | F | 5'-CGGGAAGGGAGAAGACACT-3' |
|  | R | 5'-GAGTTCCTCACGCCAACG-3' |
| Krueppel-like factor 4 (KLF4) | F | 5'-CGGGAAGGGAGAAGACACT-3' |
|  | R | 5'-GAGTTCCTCACGCCAACG-3' |
| Oct-4 | F | 5'-ATGGCATACTGTGGACCTCA-3' |
|  | R | 5'-AGCAGCTTGGCAAACTGTTC-3' |
| Elf5 | F | 5'-GTGGCATCAAGAGTCAAGACTGTC-3' |
|  | R | 5'-CTCAGCTTCTCGTACGTCATCCTG-3' |
| E-cadherin | F | 5'-AGACTTTGGTGTGGGTCAGG-3' |
|  | R | 5'-CATGCTCAGCGTCTTCTCTG-3' |
| claudin5 | F | 5'-CTTCCTGGACCACAACATCGT-3' |
|  | R | 5'-AGCGCCAGCACAGATTCATAC-3' |
| Keratin4 | F | 5'-CAAGCGTACAGCTGCAGAGAA-3' |
|  | R | 5'-TGGCCTCTAACTCCACCTTGA-3' |
| Keratin13 | F | 5'-AGTAGGCCAGGTCAATGTGGA-3' |
|  | R | 5'-TGTTCAGCTCTGCACTCTTGG-3' |
| claudin1 | F | 5'-GTGCCTGGAAGATGATGAGGT-3' |
|  | R | 5'-GGGGTCAAGGGGTCATAGAAT-3' |
| desmocollin2 | F | 5'-AAAAGTGGAGGGCAAGAGACC-3' |
|  | R | 5'-GGATCCAGGGTATGGTGATGA-3' |
|  |  |  |
| DRA,NHE2,Villin,DSC3 | Taqman primers | Applied Biosystems |
